# Round the Hospitals

**Published:** 1917-06-02

**Authors:** 


### ROUND THE HOSPITALS.

The second reading of the new Reform Bill in the House of Commons on the 22nd ultimo was made the occasion of a patriotic meeting of suffragettes at Queen's Hall, Mrs. Pankhurst being in the chair. Outsiders were charged from 7s. 6d. downwards for a seat, and the hall was three parts full. The interest centred round the announcement that Miss Christabel Pankhurst, having left Paris, would speak. A number of people, ourselves amongst them, took the occasion to attend in order to judge where Mrs. Pankhurst and her daughter and supporters stand at the moment. Miss Christabel Pankhurst has evidently been trained by masters of elocution, and her gestures include every movement of the stage witch that lovers of Shakespeare will remember. The claw-like action of two fingers and a thumb cast suddenly out to the right, followed immediately by a similar action with the two fingers and thumb of the left hand, was continually resorted to throughout the speech. Miss Pankhurst unfortunately has not lost the teetotum habit of moving her body continuously from one side to the other, and a large number of the audience in the galleries were seldom or never able to catch the whole of any sentence that she uttered.

The speech itself was carried in and placed with some ceremony upon the book-rest, and had outwardly the appearance of a something of good size in what resembled tissue paper. This mass, as the Americans would say, resented Miss Christabel's arrival by almost succeeding in precipitating itself on the floor when she first touched the easel. The speech, which occupied upwards of an hour and a half and was delivered with increasing rapidity, explained the presence of the bundle, and revealed the nature of its contents. As a speaker, Miss Christabel Pankhurst is fluent but indistinct, owing to rapidity of utterance and the teetotum habit. She often made good points and was markedly successful in dealing with interrupters, who got it straight from the shoulder and collapsed ignominiously every time. The substance of some portions of her speech was excellent in form, but the speech as a whole, if it had been cut down one-half, would have spared the audience many repetitions, and Miss Christabel could then have spoken more deliberately and slowly, and thus secured a satisfied and enlightened audience. Mrs. Pankhurst was charmingly dressed, with many brilliants, and Miss Annie Kenney was welcomed as a forcible speaker who kept to the point. The proceedings lasted so long that we had not the pleasure of hearing Mrs. Flora Drummond, who has a high reputation.

It remains to consider the impression which the platform made upon the audience. The entrance, which was delayed long after the hour fixed for the commencement of the proceedings, was unimpressive and disappointing. The cause might have lain in the delay, but whatever it was this impression made itself felt, and it could not in any sense add strength to the suffragette cause. Enthusiasm was not lacking, but in view of the new Reform Bill and the promised votes for women, if the suffragettes are to take a leading part in women's questions they will need to keep better time, to strengthen their platform and organisation, and to strengthen them quickly and much. We have always been in favour of votes for women, and have published this impression of the Queen's Hall meeting in the hope that it may prove effective for good to a cause which the majority of the nation favour.

There has grown up a considerable demand for surgical appliances and splints made of the lightest material possible. Papier mâché and vulcanite have been found invaluable in some military hospitals, and they afford great relief to warriors who have lost a limb or limbs. The *Weekly Irish Times* quotes as an example a leg bath made of papier mâché, which has a life in normal circumstances of six months and costs only 1s. 9d. A vulcanite chin-strap weighs only half an ounce, and its cost is nominal. All kinds of contrivances have been devised, and their small cost is out of all proportion to their utility. Hospital workers and others interested should visit Mulberry Walk, Chelsea, where appliances of these light descriptions are manufactured by voluntary workers, whose output has exceeded 800,000. Nevertheless the demand for such articles is far in excess of the supply, and the Irish women, led by Mrs. Hignett, are organising a vacant house in Merrion Square, Dublin, placed at the disposal of the committee, to enable papier mâché and vulcanite appliances to be manufactured there. We wish the venture every success, and shall be glad to hear of its progress from time to time.

Miss Rundle, the secretary of the College of Nursing, and Miss Alsop, matron of the Kensington Infirmary and honorary secretary of the Poor-Law Infirmary Matrons' Association, addressed a meeting at the Norfolk and Norwich Hospital on Tuesday, May 22, on behalf of the College of Nursing. Dr. Barton, the chairman of the nursing committee, was in the chair, and representatives of hospitals, Poor-Law institutions, and nursing associations in the district were present. The Norfolk and Norwich Hospital is one of those which from the first has appreciated the value such an organisation as the College cannot fail to be to the nursing profession. The College hopes to help also, not only the large hospitals, but the small and special ones and the Poor-Law training schools. In regard to the latter, Miss Alsop pointed out that on the establishment of the one-portal examination there could be no room for the suggestion sometimes made that the Poor-Law trained nurse was inferior to the hospital trained. When the College Register is published it is hoped it will include a record of all extra qualifications possessed by nurses, such as midwifery, massage, sanitary certificate, etc.

**ROUND THE HOSPITALS—*(continued).***

THE memorial projects to Edith Cavell are so numerous that it is satisfactory to learn that in the capital city of her native county strength is to be given to the local memorials by the amalgamation of the two proposed to be established. In Norwich the two schemes were for the erection of a memorial statue from funds provided through a shilling fund raised by the late Lord Mayor of Norwich, Dr. Gordon Munn, and for the enlargement of the work carried on by the Norwich District Nurses' Association. It is proposed that the statue, the commission for which Mr. Pegram, the sculptor of the Sir Thomas Browne memorial has accepted, shall be erected opposite the District Nurses' Association's premises on Tombland, a site which is held to be the finest in Norwich for the memorial statue, being opposite the main cathedral gate, at the old historic centre of the city. Its immediate contiguity to the premises of the District Nurses' Association is a happy circumstance which cannot fail to give added force to the memorial character of such a statue and secure the unity of the two schemes. A sum of £2,500 is appealed for, to purchase and equip the freehold of the premises on Tombland now occupied by the Association, together with an adjoining property, and already certain subscriptions have been promised. The property is to be known as "The Cavell Home" and we can imagine no fitter memorial to the memory of one "who lived for humanity and died for her country" than such an amalgamation of the Norwich schemes designed to perpetuate the memory of Britain's martyred nurse.

---

AN important feature of the June issue of *The Modern Hospital* is to be a paper prepared by Miss Alice F. Bell, under the auspices of the department of nursing, Teachers' College, Columbia University, on the standardisation of records in training-schools for nurses. This is work for which there has long been a crying need, and the system outlined in this paper may be of epoch-making importance in nursing education.

---

*** It is our invariable practice to pay for all items of news which we may publish. The minimum payment is 5s. Paragraphs of about twenty lines in length—that is to say, of 150 words or less—are preferred. In this section during the war we propose to give 10s. to the correspondent who may send us in any week the best incident connected with hospital life and work. In this way we hope to afford to all practical aid and encouragement; while those who reciprocate will be able to render us invaluable service. Contributions should be addressed to The Editor, THE HOSPITAL, 28 and 29 Southampton Street, Strand, London, W.C., and, to be in time for the current week's issue, should reach him by the first post on Mondays.

### The Supply of Nurses Committee.

THE Advisory Committee, appointed by the Army Council to inquire into the supply of nurses, issued an interim report, dated November 14, 1916, which has now been placed at the disposal of the two Houses of Parliament and such other persons has may have applied to the Secretary of the War Office for a copy. We have still to await the final report of the Committee, without which and until we receive it the best course appears to be to publish the following summary of the recommendations:—

TRAINED NURSES.

1. That no more trained nurses can be obtained for military hospitals from institutions managed by the public health, lunacy, or Poor-Law authorities, with the exception of the institutions recognised by the Local Government Board as training-schools, or from district nurses, school nurses, or health visitors.
2. Small auxiliary hospitals should be restricted to the treatment of convalescent cases. No new hospital should be set up with less than forty beds.
3. Secure all available probationers from training-schools as they complete their full training.
4. Aim at a maximum proportion of one trained nurse to fourteen beds in ordinary hospitals, and one to six beds in private hospitals for officers.
5. Appeal to private nurses, their employers and associations, to volunteer for war work, offering compensation where there is serious financial loss, and securing a return of the nurses to their former position at the close of the war.
6. Ask the Dominions if they can provide an additional number of trained nurses, but abstain from recruiting in the United States* or other foreign countries.
7. In a case of emergency employ probationers (including V.A.D. members) as nurses under a sister where they are approved by the matron-in-charge.
8. Increase the staff of all military hospitals, so as to ensure regular leave and more relaxation where required.

* The report is dated January 24, 1917—before the entry of the United States into the war.

9. Give a bonus for every six months of service in military hospitals, with an increment of £1 for each period of six months after the first—to be paid on termination of service.
10. Provide better pensions in cases of permanent breakdown due to war service.

FOR TRAINED AND UNTRAINED NURSES.

11. Provide transport in covered conveyance where nurses have to live at a distance from the hospitals where they work, and in camp hospitals.
12. Insist on better accommodation for nurses where it is now inadequate. Provide a matron-inspector to look into the conditions of all war hospitals.
13. Promise recognition of satisfactory service for all nurses working in all hospitals receiving sick and wounded soldiers.
14. Provide in all cases travelling expenses at reduced fares.

FOR UNTRAINED NURSES.

15. Provide cost of medical examination for all probationers, including V.A.D. members.
16. Relieve them of expense as regards uniform.
17. Furnish additional allowance where required for necessary expenses of members in V.A.D. hospitals.
18. Grade V.A.D. members so as to distinguish those who have had thirteen months of approved service.
19. Establish hostels where V.A.D. members may reside and hear lectures, and from which they can go for daily training in civil hospitals.
20. Appoint a matron-inspector of V.A.D. auxiliary and private hospitals for each Command in the United Kingdom, whose reports should be submitted through the Commands to the War Office for final action.
21. Make arrangements that the trained matron should be in charge of the nursing staff in V.A.D. auxiliary hospitals.

GENERAL RECOMMENDATIONS.

22. Bring all the nursing authorities into touch by establishment of one central committee to act as a clearing-house during war time.
23. Encourage the making of a census of all women suitable for nursing, and willing to undertake it for the period of the war.

